# Supplementary material for: Antibiotic Use and Antibiotic Resistance: Public Awareness Survey in the Republic of Cyprus
Source: Antibiotics (Basel). 2020 Oct 30;9(11):759. doi: 10.3390/antibiotics9110759 (PMC7692346; doi:10.3390/antibiotics9110759)
Supplement: Supplementary file 1 [file antibiotics-09-00759-s001.pdf]

ΔΗΜΟΓΡΑΦΙΚΑ ΣΤΟΙΧΕΙΑ

1. Καταγράψτε το φύλο σας
   a. Άντρας
   b. Γυναίκα

2. Ηλικία
   a. 16-18
   b. 19-24
   c. 25-34
   d. 35-44
   e. 45-54
   f. 55-64
   g. 65+

3. Σε ποιά επαρχία ζείτε;
   a. Λευκωσία
   b. Λάρνακα
   c. Λεμεσός
   d. Πάφος
   e. Αμμόχωστος

4. Ποιο από αυτά περιγράφει καλύτερα πού ζείτε;
   a. Πόλη
   b. Προάστιο πόλης
   c. Αγροτική - έξω από μια πόλη π.χ. χωριό / εξοχή / περιοχή καλλιέργειας

5. Ποιος είναι ο υψηλότερος βαθμός ή το επίπεδο του σχολείου που έχετε ολοκληρώσει;

   Εάν είστε ήδη εγγεγραμμένοι, λαμβάνεται ως ο υψηλότερος βαθμός.

   a. Δεν ολοκληρώθηκε καμία εκπαίδευση
   b. Απόφοιτος δημοτικού
   c. Απόφοιτος γυμνασίου
   d. Απόφοιτος λυκείου
   e. Πτυχίο πανεπιστημίου
   f. Μεταπτυχιακό
   g. Διδακτορικό

6. Ποιο από τα παρακάτω περιγράφει καλύτερα το συνολικό εισόδημα του νοικοκυριού σας, προ φόρων;
   a. <10,000
   b. 10,000-20,000
   c. 20,000-30,000
   d. >30,000

7. Ποιο από αυτά περιγράφει καλύτερα τη σύνθεση του νοικοκυριού σας
    a. 1 ενήλικας
    b. 1 ενήλικας και τουλάχιστον 1 παιδί κάτω των 16 ετών
    c. Έγγαμος/η - μόνο ενήλικες
    d. Έγγαμος/η / και τουλάχιστον 1 παιδί κάτω των 16 ετών
    e. Πολλά άτομα ηλικίας 16+ μόνο
    f. Πολλαπλοί ενήλικες ηλικίας 16+ ετών και τουλάχιστον 1 παιδί κάτω των 16 ετών

ΚΥΡΙΟ ΕΡΩΤΗΜΑΤΟΛΟΓΙΟ

1. Πότε πήρατε τελευταία φορά αντιβιοτικά;
    a. Τον τελευταίο μήνα
    b. Τους τελευταίους 6 μήνες
    c. Τον τελευταίο χρόνο
    d. Πάνω από ένα χρόνο πριν
    e. Ποτέ
    f. Δεν μπορώ να θυμηθώ

Εάν απαντήσατε "Ποτέ", πηγαίνετε κατευθείαν στην Ερώτηση 5.

2. Πήρατε τα αντιβιοτικά (ή μια συνταγή για αυτά) από γιατρό;
    a. Ναι
    b. Όχι
    c. Δεν μπορώ να θυμηθώ

3. Λάβατε συμβουλές από γιατρό, νοσοκόμα ή φαρμακοποιό για το πώς να τα πάρετε;
    a. Ναι, έλαβα συμβουλές για το πώς να τα πάρω (π.χ. με φαγητό, για 7 ημέρες)
    b. Όχι
    c. Δεν μπορώ να θυμηθώ

4. Απο πού πήρατε τα αντιβιοτικά;
    a. Φαρμακείο
    b. Το διαδίκτυο
    c. Φίλος ή μέλος της οικογένειας
    d. Είχα φυλάξει από μια προηγούμενη φορά
    e. Κάπου αλλού
    f. Δεν μπορώ να θυμηθώ

ΓΝΩΣΕΙΣ ΓΙΑ ΤΑ ΑΝΤΙΒΙΟΤΙΚΑ

5. Πότε νομίζετε ότι πρέπει να σταματήσετε να παίρνετε αντιβιοτικά όταν έχετε αρχίσει μια θεραπεία;
    a. Όταν αισθάνεστε καλύτερα
    b. Όταν έχετε πάρει όλα τα αντιβιοτικά σύμφωνα με τις οδηγίες
    c. Δεν ξέρω

6. Πιστεύετε ότι αυτή η δήλωση είναι «αληθής» ή «ψευδής»;
   "Είναι εντάξει να χρησιμοποιείτε αντιβιοτικά που έχουν δοθεί σε έναν φίλο ή μέλος της οικογένειας, εφ 'όσον αυτά χρησιμοποιήθηκαν για τη θεραπεία της ίδιας ασθένειας "
    a. Αληθής
    b. Ψευδής
    c. Δεν ξέρω

7. Πιστεύετε ότι αυτή η δήλωση είναι «αληθής» ή «ψευδής»;
   "Είναι εντάξει να αγοράσετε τα ίδια αντιβιοτικά ή να τα ζητήσετε από γιατρό, αν είστε άρρωστοι και αυτα σας βοήθησαν να βελτιωθείτε όταν είχατε τα ίδια συμπτώματα πριν "
    a. Αληθής
    b. Ψευδής
    c. Δεν ξέρω

8. Ποιές απο αυτές τις καταστάσεις πιστεύετε πως μπορούν να αντιμετωπιστούν με αντιβιοτικά;
    a. HIV / AIDS
    b. Βλεννόρροια
    c. Η λοίμωξη της ουροδόχου κύστης ή η λοίμωξη του ουροποιητικού συστήματος (UTI)
    d. Διάρροια
    e. Κρύωμα και γρίπη
    f. Πυρετός
    g. Ελονοσία
    h. Ιλαρά
    i. Λοίμωξη του δέρματος ή της πληγής
    j. Πονόλαιμος
    k. Σωματικοί πόνοι Πονοκέφαλοι

ΓΝΩΣΕΙΣ ΓΙΑ ΤΗΝ ΑΝΤΙΒΙΟΤΙΚΗ ΑΝΤΟΧΗ

9. Έχετε ακούσει κάποιο από τους ακόλουθους όρους
    a. Αντιβιοτική αντοχή
    b. Αντιμικροβιακή αντοχή
    c. Βακτήρια ανθεκτικά στα αντιβιοτικά

10. Απαντήστε εάν επιλέχθηκε η επιλογή "Αντιβιοτική αντοχή" στην ερώτηση 9.
    Πού ακούσατε για τον όρο: «Αντιβιοτική αντοχή»;
    a. Γιατρός ή νοσοκόμα
    b. Φαρμακοποιός
    c. Μέλος της οικογένειας ή φίλος (συμπεριλαμβανομένων των κοινωνικών μέσων ενημέρωσης)
    d. Μέσα μαζικής ενημέρωσης (εφημερίδα, τηλεόραση, ραδιόφωνο)
    e. Ειδική καμπάνια
    f. Αλλα
    g. Δεν μπορώ να θυμηθώ

11. Απαντήστε εάν επιλέχθηκε η επιλογή «Αντιμικροβιακή αντοχή» στην ερώτηση 9.
    Πού ακούσατε για τον όρο: «Αντιμικροβιακή αντοχή»;

    a. Γιατρός ή νοσοκόμα
    b. Φαρμακοποιός
    c. Μέλος της οικογένειας ή φίλος (συμπεριλαμβανομένων των κοινωνικών μέσων ενημέρωσης)
    d. Μέσα μαζικής ενημέρωσης (εφημερίδα, τηλεόραση, ραδιόφωνο)
    e. Ειδική καμπάνια
    f. Αλλα
    g. Δεν μπορώ να θυμηθώ

12. Απαντήστ εάν επιλέχθηκε η επιλογή " Βακτήρια ανθεκτικά στα αντιβιοτικά"στην ερώτηση 9.
    Πού ακούσατε για τον όρο: " Βακτήρια ανθεκτικά στα αντιβιοτικά";

    a. Γιατρός ή νοσοκόμα
    b. Φαρμακοποιός
    c. Μέλος της οικογένειας ή φίλος (συμπεριλαμβανομένων των κοινωνικών μέσων ενημέρωσης)
    d. Μέσα μαζικής ενημέρωσης (εφημερίδα, τηλεόραση, ραδιόφωνο)
    e. Ειδική καμπάνια
    f. Αλλα
    g. Δεν μπορώ να θυμηθώ

13. Παρακαλείσθε να αναφέρετε εάν πιστεύετε ότι οι ακόλουθες δηλώσεις είναι «αληθείς» ή «ψευδείς»

    a. Η αντοχή στα αντιβιοτικά εμφανίζεται όταν το σώμα σας γίνεται ανθεκτικό στα αντιβιοτικά, ετσι δεν λειτουργούν σωστά. **Α / Ψ**
    b. Πολλές λοιμώξεις καθίστανται όλο και περισσότερο ανθεκτικές στη θεραπεία με αντιβιοτικά **Α / Ψ**
    c. Εάν τα βακτήρια είναι ανθεκτικά στα αντιβιοτικά, μπορεί να είναι πολύ δύσκολο ή αδύνατο να αντιμετωπιστούν οι λοιμώξεις που προκαλούν **Α / Ψ**
    d. Η αντοχή στα αντιβιοτικά είναι ένα ζήτημα που μπορεί να επηρεάσει εμένα ή την οικογένειά μου **Α / Ψ**
    e. Η αντοχή στα αντιβιοτικά είναι θέμα σε άλλες χώρες αλλά όχι εδώ **Α / Ψ**
    f. Η αντοχή στα αντιβιοτικά είναι πρόβλημα μόνο για τους ανθρώπους που λαμβάνουν τακτικά αντιβιοτικά **Α / Ψ**
    g. Τα βακτήρια που είναι ανθεκτικά στα αντιβιοτικά μπορούν να εξαπλωθούν από άτομο σε άτομο **Α / Ψ**
    h. Οι ανθεκτικές στα αντιβιοτικά, λοιμώξεις, καθιστούν ιατρικές επεμβάσεις, όπως χειρουργική επέμβαση, μεταμοσχεύσεις οργάνων και την θεραπεία του καρκίνου, πολύ πιο επικίνδυνες **Α / Ψ**

14. Πιστεύετε ότι τα αντιβιοτικά χρησιμοποιούνται ευρέως στη γεωργία (συμπεριλαμβανομένων και των ζώων που χρησιμοποιούνται για την παραγωγή τροφίμων) στη χώρα σου?
    a. Ναι
    b. Όχι
    c. Δεν ξέρω

		Συμφωνώ Πολύ	Συμφωνώ Ελαφρώς	Ούτε συμφωνώ ούτε διαφωνώ	Διαφωνώ Ελαφρώς	Διαφωνώ Πολύ
1	Οι άνθρωποι πρέπει να χρησιμοποιούν αντιβιοτικά μόνο όταν συνταγογραφούνται από γιατρό	5	4	3	2	1
2	Οι αγρότες πρέπει να δίνουν λιγότερα αντιβιοτικά σε ζώα που παράγουν τρόφιμα	5	4	3	2	1
3	Οι άνθρωποι δεν πρέπει να κρατούν αντιβιοτικά και να τα	5	4	3	2	1

	χρησιμοποιούν αργότερα για άλλες ασθένειες					
4	Οι γονείς πρέπει να εξασφαλίζουν όλους τους εμβολιασμούς των παιδιών τους	5	4	3	2	1
5	Οι άνθρωποι πρέπει να πλένουν τα χέρια τους τακτικά	5	4	3	2	1
6	Οι γιατροί πρέπει να συνταγογραφούν αντιβιοτικά μόνο όταν χρειάζονται	5	4	3	2	1
7	Οι κυβερνήσεις θα πρέπει να επιβραβεύουν την ανάπτυξη νέων αντιβιοτικών	5	4	3	2	1
8	Οι φαρμακευτικές εταιρείες πρέπει να αναπτύξουν νέα αντιβιοτικά	5	4	3	2	1

		Συμφωνώ Πολύ	Συμφωνώ Ελαφρώς	Ούτε συμφωνώ ούτε διαφωνώ	Διαφωνώ Ελαφρώς	Διαφωνώ Πολύ
1	Η αντοχή στα αντιβιοτικά είναι ένα από τα μεγαλύτερα προβλήματα στον κόσμο	5	4	3	2	1
2	Οι ιατροί και οι εμπειρογνώμονες θα λύσουν το πρόβλημα της αντοχής στα αντιβιοτικά πριν γίνει πολύ σοβαρό	5	4	3	2	1
3	Ο καθένας πρέπει να αναλάβει ευθύνη	5	4	3	2	1

	χρησιμοποιώντας αντιβιοτικά υπεύθυνα					
4	Δεν υπάρχουν πολλά που άνθρωποι σαν εμένα μπορούν να κάνουν για να σταματήσει η αντοχή στα αντιβιοτικά	5	4	3	2	1
5	Ανησυχώ για τον αντίκτυπο που η αντοχή στα αντιβιοτικά θα έχει πάνω στην υγεία μου και αυτή της οικογένειάς μου	5	4	3	2	1
6	Εφόσον λαμβάνω τα αντιβιοτικά μου σωστά, δεν είμαι σε κίνδυνο να προσβληθώ από ανθεκτική στα αντιβιοτικά λοίμωξη	5	4	3	2	1

Σας δίνω την άδειά μου να επικοινωνήσετε μαζί μου στο μέλλον για να ξανακάνω αυτό το ερωτηματολόγιο.-

E-mail:

Τηλέφωνο:
